# Multiple Independent Genetic Factors at *NOS1AP* Modulate the QT Interval in a Multi-Ethnic Population

**DOI:** 10.1371/journal.pone.0004333

**Published:** 2009-01-30

**Authors:** Dan E. Arking, Amit Khera, Chao Xing, W. H. Linda Kao, Wendy Post, Eric Boerwinkle, Aravinda Chakravarti

**Affiliations:** 1 McKusick-Nathans Institute of Genetic Medicine, Johns Hopkins University School of Medicine, Baltimore, Maryland, United States of America; 2 Donald W. Reynolds Cardiovascular Clinical Research Center, University of Texas Southwestern Medical Center, Dallas, Texas, United States of America; 3 Division of Cardiology, University of Texas Southwestern Medical Center, Dallas, Texas, United States of America; 4 McDermott Center for Human Growth and Development, University of Texas Southwestern Medical Center, Dallas, Texas, United States of America; 5 Department of Epidemiology, Johns Hopkins Bloomberg School of Public Health, Baltimore, Maryland, United States of America; 6 Department of Medicine, Johns Hopkins University School of Medicine, Baltimore, Maryland, United States of America; 7 Human Genetics Center and Division of Epidemiology, The University of Texas, Houston, Texas, United States of America; Innsbruck Medical University, Austria

## Abstract

Extremes of electrocardiographic QT interval are associated with increased risk for sudden cardiac death (SCD); thus, identification and characterization of genetic variants that modulate QT interval may elucidate the underlying etiology of SCD. Previous studies have revealed an association between a common genetic variant in *NOS1AP* and QT interval in populations of European ancestry, but this finding has not been extended to other ethnic populations. We sought to characterize the effects of *NOS1AP* genetic variants on QT interval in the multi-ethnic population-based Dallas Heart Study (DHS, n = 3,072). The SNP most strongly associated with QT interval in previous samples of European ancestry, rs16847548, was the most strongly associated in White (P = 0.005) and Black (P = 3.6×10^−5^) participants, with the same direction of effect in Hispanics (P = 0.17), and further showed a significant SNP × sex-interaction (P = 0.03). A second SNP, rs16856785, uncorrelated with rs16847548, was also associated with QT interval in Blacks (P = 0.01), with qualitatively similar results in Whites and Hispanics. In a previously genotyped cohort of 14,107 White individuals drawn from the combined Atherosclerotic Risk in Communities (ARIC) and Cardiovascular Health Study (CHS) cohorts, we validated both the second locus at rs16856785 (P = 7.63×10^−8^), as well as the sex-interaction with rs16847548 (P = 8.68×10^−6^). These data extend the association of genetic variants in *NOS1AP* with QT interval to a Black population, with similar trends, though not statistically significant at P<0.05, in Hispanics. In addition, we identify a strong sex-interaction and the presence of a second independent site within *NOS1AP* associated with the QT interval. These results highlight the consistent and complex role of *NOS1AP* genetic variants in modulating QT interval.

## Introduction

The electrocardiographic QT interval is a measure of cardiac repolarization, and based on observations in both long and short QT syndrome cases [1], as well as in population-based cohorts [2]–[8], serves as a useful marker of risk for sudden cardiac death (SCD). A genome-wide association study identified a common genetic variant in *NOS1AP*, rs10494366, as being associated with altered QT interval in individuals of European ancestry [9]. This finding has been subsequently replicated in additional populations of European ancestry [10]–[14], including reports of a stronger effect in women [15]. However, in the one study that examined African Americans, no significant effect was observed [16]. In a large population-based study, we have refined the location of the association signal for the QT interval in Whites and also demonstrated that the variant most strongly associated with prolongation of the QT interval, rs16847548, was also associated with increased risk for SCD [17]. However, no association with either QT interval or SCD was observed in Blacks in that study. Nevertheless, given the overwhelming evidence for association in populations of European ancestry and potential gender specific effects, we sought to further explore the role of genetic variants in *NOS1AP* across multiple ethnic backgrounds. We hypothesized that there is allelic heterogeneity at this *NOS1AP* locus.

## Results

**Figure 1:**
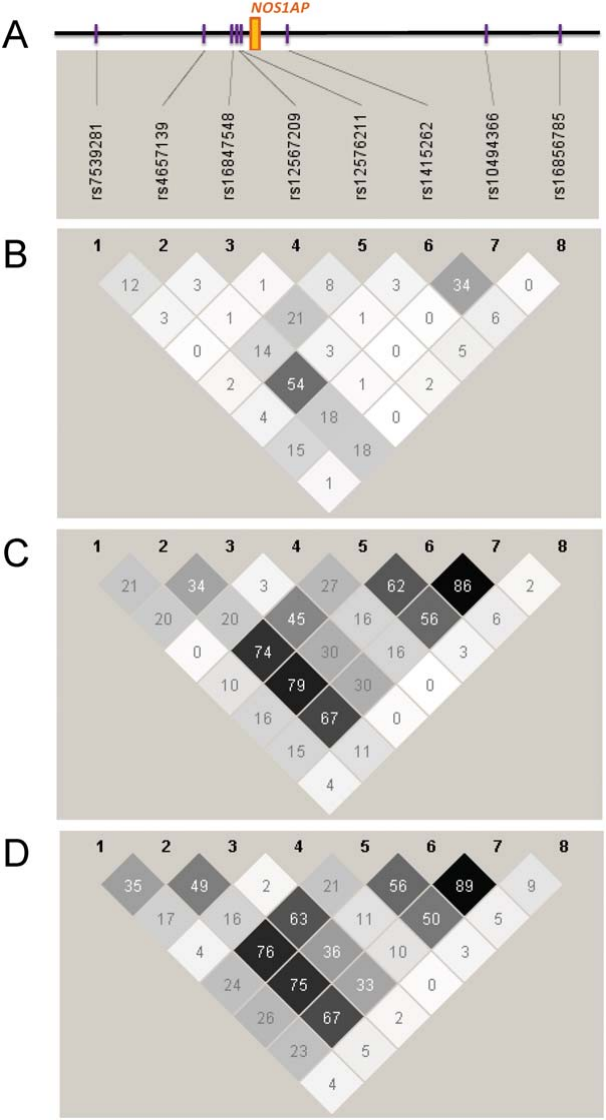
Comparison of linkage disequilibrium (LD) patterns across ethnic groups. A) Schematic of the *NOS1AP* locus, with exon 1 in orange, and genotyped SNPs in purple. The region spans 92 kb. Linkage disequilibrium (LD) is defined as the pair-wise correlation between SNPs (measured as R-square) at the top left and the top right sides of the diamond. The degree of shading represents the magnitude and significance of the pair-wise LD, with a black to white gradient reflecting higher to lower LD values; see http://www.broad.mit.edu/mpg/haploview/ for further details. B) Non-Hispanic Blacks; C) Hispanics; D) Non-Hispanic Whites.

The Dallas Heart Study (DHS) is a multi-ethnic probability-based, population study [18], with 1,506 non-Hispanic Blacks, 942 non-Hispanic Whites, and 501 Hispanics available for the analysis presented here. Eight SNPs chosen to tag the linkage disequilibrium (LD) block previously associated with QT interval (based on LD in the HapMap CEU samples), including the SNP associated with SCD [17], were genotyped in all individuals. Thirteen additional individuals were excluded due to excessive missing genotype data (<50% complete); all SNPs showed minor missing data (≥99% complete) with no strong deviation from Hardy-Weinberg equilibrium (P<0.005) in any of the populations. The non-Hispanic White and Hispanic participants had similar patterns of LD and similar allele frequencies, whereas the non-Hispanic Black participants demonstrated significantly less LD, with complementary minor alleles for 6 of 8 SNPs (Figure 1, Table 1).

**Table 1:** Summary of results for association of *NOS1AP* SNPs with QT interval stratified by ethnicity.

SNP	Position	A1	A2	Non-Hispanic Blacks (n = 1,497)				Hispanics (n = 499)				Non-Hispanic Whites (n = 940)			
				A1 Freq	β	SE	P	A1 Freq	β	SE	P	A1 Freq	β	SE	P
rs7539281	158739692	A	G	0.60	0.18	0.63	0.77	0.29	0.66	0.90	0.4639	**0.26**	**1.81**	**0.66**	**0.006**
rs4657139	158761565	A	T	0.88	1.59	0.90	0.08	0.42	0.98	0.85	0.2476	**0.34**	**1.21**	**0.60**	**0.043**
rs16847548	158766932	C	T	**0.18**	**3.22**	**0.78**	**3.58E-05**	0.20	1.47	1.07	0.1706	**0.20**	**2.57**	**0.72**	**0.0004**
rs12567209	158768137	A	G	0.08	0.08	1.13	0.95	0.12	−1.00	1.23	0.4175	0.08	−0.45	1.09	0.68
rs12576211	158768181	T	G	**0.51**	**1.83**	**0.61**	**0.003**	0.35	0.01	0.87	0.9919	**0.29**	**1.81**	**0.63**	**0.005**
rs1415262	158777793	C	G	0.81	1.13	0.76	0.14	0.43	1.11	0.85	0.1931	0.35	0.81	0.60	0.18
rs10494366	158817343	G	T	0.62	1.22	0.63	0.05	0.41	1.52	0.84	0.07228	0.36	0.72	0.60	0.23
rs16856785	158831945	C	G	**0.61**	**1.60**	**0.62**	**0.01**	0.10	0.58	1.30	0.6573	0.10	0.95	0.97	0.33

A1 refers to the minor allele in the CEU HapMap population. **Bold** signifies P-values<0.05. Genomic position is given relative to Build35 of the Human Genome. β is the effect size under an additive genetic model. SE = standard error.

The QT interval is highly correlated with heart rate (RR interval), age, and sex at the population level, and thus we performed multiple linear regression analyses separately in each ethnic population to adjust for these factors, and analyzed the residual QT under an additive genetic model (Table 1). In non-Hispanic Whites, the results mirrored our previous findings, with the strongest association observed for rs16847548, with each allele associated with an increase in the QT interval of 2.57 ms (P = 0.0004). While no nominally significant SNPs were identified in Hispanics, all SNPs exhibited the same direction of effect as in non-Hispanic Whites. Strikingly, and in contrast to previous reports, rs16847548 was strongly associated with QT interval in non-Hispanic Blacks, with each allele associated with a prolongation of the QT interval by 3.22 ms (P = 3.58×10^−5^). Another novel finding was that rs16856785, which is uncorrelated with rs16847548 (r^2^<0.01), was also associated with QT interval in non-Hispanic Blacks (+1.60 ms, P = 0.01). While not significant in Hispanics or non-Hispanic Whites, the direction of effect observed for rs16856785 was the same (+0.58 ms and +0.95 ms, respectively). To formally test for independent effects of these two SNPs, we performed a forward stepwise regression in each ethnic group, first including the most strongly associated SNP and then sequentially adding SNPs in order of strength of association, only retaining them in the model if the P-values were <0.05. Only rs16847548 was retained in Hispanics and non-Hispanic Whites, but both rs16847548 and rs16856785 were significant in non-Hispanic Blacks indicating that these 2 SNPs independently influence QT interval in non-Hispanic Blacks (Table 2). To determine whether population stratification was influencing our results, we adjusted for both global and local ancestries as inferred by 2,270 ancestry informative markers using ANCESTRYMAP [19], and our findings were unchanged. Comparing non-Hispanic Blacks homozygous at both SNPs for the QT lengthening allele to non-Hispanic Blacks homozygous for the complementary alleles revealed a 13.9±4.5 ms difference in QT interval.

**Table 2:** Demonstration of independent effects for rs16847548 and rs16856785 on QT interval stratified by ethnicity.

Model	SNP	A1	A2	Non-Hispanic Blacks (n = 1,497)				Non-Hispanic Whites (n = 940)				ARIC/CHS Whites (n = 14,107)			
				A1 Freq	β	SE	P	A1 Freq	β	SE	P	A1 Freq	β	SE	P
Single SNP	rs16847548	C	T	**0.18**	**3.22**	**0.78**	**3.58E-05**	**0.20**	**2.57**	**0.72**	**0.0004**	**0.22**	**2.42**	**0.22**	**<2.00E-16**
	rs16856785	C	G	**0.61**	**1.60**	**0.62**	**0.01**	0.10	0.95	0.97	0.33	**0.09**	**2.11**	**0.32**	**3.94E-11**
Multi-SNP	rs16847548	C	T	**0.18**	**3.22**	**0.78**	**3.66E-05**	**0.20**	**2.62**	**0.73**	**0.0004**	**0.22**	**2.22**	**0.23**	**<2.00E-16**
	rs16856785	C	G	**0.61**	**1.60**	**0.62**	**0.01**	0.10	0.24	0.99	0.81	**0.09**	**1.74**	**0.32**	**7.63E-08**

Single SNP indicates a regression model with rs16847548 OR rs16856785, and multi-SNP indicates both SNPs are in the model. A1 refers to the minor allele in the CEU HapMap population. **Bold** signifies P-values<0.05. Genomic position is given relative to Build35 of the Human Genome. β is the effect size under an additive genetic model. SE = standard error.

**Figure 2:**
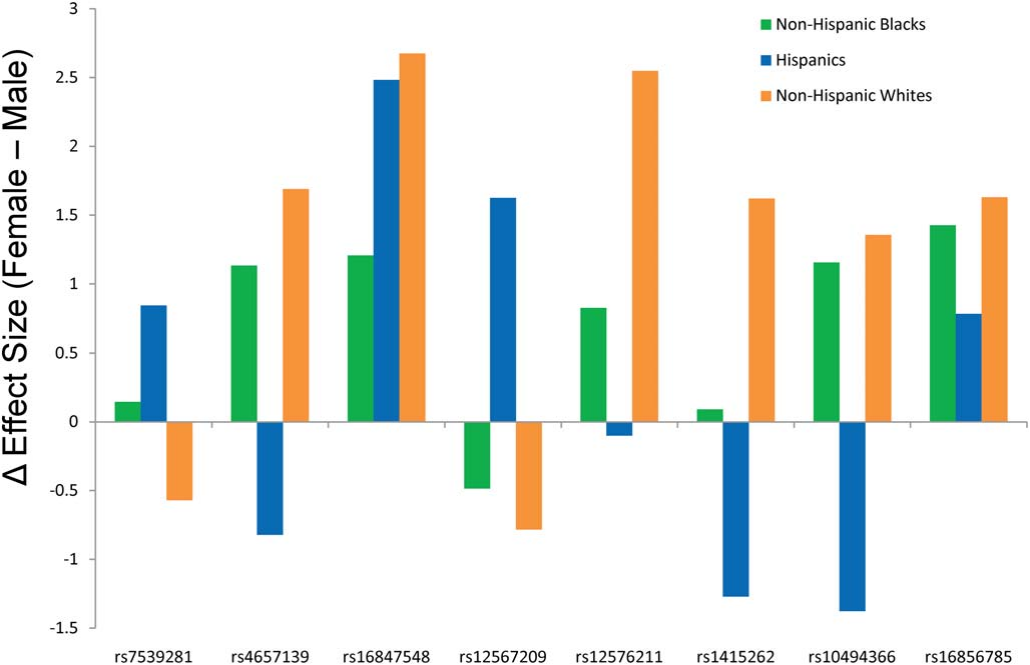
Sex-stratified analysis of *NOS1AP* SNPs for association with QT interval. The “Δ Effect Size” indicates the effect size in males subtracted from that observed in females. Hence a positive value indicates a larger effect in women. Effect sizes are in ms, and are per allele under an additive genetic model.

When stratified by sex, we observed stronger effects for *NOS1AP* variants in women (Figure 2), with a consistent effect across all 3 ethnic groups, and an average addition of +2.18 ms in the effect estimate relative to men for rs16847548. To increase our power to test for a potential sex interaction, we leveraged the similarity in effect sizes and allele frequencies for rs16847548 across all three ethnic groups, and performed a joint analysis while adjusting for ethnicity. Across all samples, we observed genome-wide significance for rs16847548 alone (P = 4.1×10^−8^), while incorporating sex and an interaction term for rs16847548 and sex into the regression model indicated a statistically significant stronger effect in women (one-sided P = 0.027 for the interaction term). No significant interaction was observed for rs16856785.

To confirm both the sex interaction and independence of the effects of rs1684758 and rs16856785, we reanalyzed data from the combined Atherosclerotic Risk in Communities (ARIC) [20] and Cardiovascular Health Study (CHS) [21] cohorts. QT interval was corrected for age, sex, RR interval, and study using linear regression. We confined our analyses to the white individuals (N = 14,107), as no association between *NOS1AP* variants and QT interval was previously observed in the black individuals in those samples [17]. Incorporating both rs16847548 and rs16856785 into a linear regression model revealed that both were significantly associated with QT interval (P<2×10^−16^ and P = 7.63×10^−8^, respectively), with similar effect sizes as observed for the DHS (+2.22 ms and +1.74 ms, respectively), confirming that these two SNPs are independently associated with QT interval (Table 2). The interaction between rs16847548 and sex in Whites was also highly significant (+2.00 ms, P = 8.68×10^−6^ for the interaction term), whereas no sex interaction was observed for rs16856785 (P = 0.31).

## Discussion

Previous studies have focused on identifying and validating a single functional variant in *NOS1AP* associated with QT interval in populations of European ancestry. Not only do our data extend the findings of association between QT interval and *NOS1AP* to non-Hispanic Blacks, but we further identify a second independent association within *NOS1AP*. Intriguingly, the first locus, represented by rs16847548 shows a strong sex-interaction, whereas the second locus, represented by rs16856785 does not. Resequencing of all exons in *NOS1AP* (ref. 9 and unpublished data) has not identified any missense mutations that explain these results, suggesting that the functional variants associated with these SNPs are likely regulatory in nature. Several limitations to the current study need to be acknowledged. First, the 8 SNPs were chosen based on their ability to tag SNPs in a Caucasian population, and thus do not fully screen the region in Blacks. Second, we were underpowered in the Hispanics, with only 55% power to observe a significant effect (P<0.05) at rs16847548, assuming equal effect to that observed in non-Hispanic Whites. Third, we have focused upon a single LD block within *NOS1AP*, whereas it is quite possible that additional variants outside of this LD block also modulate QT interval, and thus we are likely underestimating the overall impact of this gene on QT interval. In summary, these data highlight the complex genetic architecture underlying common traits even within a single gene, and specifically, begin to elucidate how *NOS1AP* genetic variants modulate QT interval.

## Methods

### Ethics Statement

The DHS was approved by the Institutional Review Board of the University of Texas Southwestern Medical Center at Dallas, TX, and conducted in accordance with institutional guidelines; all participants provided written informed consent. All protocols for both ARIC and CHS were approved by each field center's institutional review board and conducted in accordance with institutional guidelines; all participants provided written informed consent.

### Study Populations

The Dallas Heart Study (DHS) is a multi-ethnic probability-based, population study. Complete details of the DHS design have been described elsewhere [18]. Briefly, a stratified random sample of Dallas County residents age 18–65 was obtained from a pool of 841,943 eligible subjects using the U.S. Postal Service Delivery Sequence File, with deliberate oversampling of African Americans. From 10 geographic strata of different ethnic compositions, random samples totaling 15,088 addresses were identified, and 7,586 eligible patients were selected, including at most 1 subject from each address. Of these, 6,101 subjects ages 18–65 (52% non-Hispanic Black, 29% non-Hispanic White, 17% Hispanic, and 2% other) were recruited to participate in 3 sequential visits. Visit 1 included an in-home survey and anthropometric measurements (n = 6101); visit 2- blood and urine samples (n = 3399, ages 30–65); visit 3- imaging tests and electrocardiograms (n = 3072). Demographic variables, body-mass index, and blood pressure were similar between subjects completing the initial visit, and Visits 2 and 3 [18]. After removing individuals with no QT interval data, QRS duration >120 ms, or ethnicity listed as “other”, 1,506 non-Hispanic Blacks, 942 non-Hispanic Whites, and 501 Hispanics were available for analysis.

The ARIC study and CHS are both population-based prospective cohort studies of cardiovascular disease. The ARIC Study includes 15,792 persons aged 45–64 years at baseline (1987–89), randomly chosen from four US communities [20]. ARIC cohort members completed four clinic examinations, conducted approximately three years apart between 1987 and 1998. CHS includes 5,888 participants >65 years of age identified from four U.S. communities using Medicare eligibility lists. The original cohort included 5201 participants recruited in 1989–1990 and 687 additional subjects were recruited in 1992–1993 to enhance the racial/ethnic diversity of the cohort [21]. Clinic examinations for both ARIC and CHS participants included assessment of cardiovascular risk factors, self-reported medical family history, employment and educational status, diet, physical activity, co-morbidities, and clinical and laboratory measurements. The following exclusion criteria, were applied to obtain the final sample for the present analysis: poor quality DNA (samples with <75% of genotypes called), no QT interval data, QRS duration >120 ms, self-described ethnicity other than White [17]. After these exclusions, 14,107 individuals were available for analysis.

### Assessment of QT interval

In the DHS, participants presented in a fasting state for a detailed clinic visit which included electrocardiography. They were placed in a supine position with application of standard 12-lead electrodes, and after a brief period of relaxation, a 10 second digital electrocardiogram was recorded using the GE Marquette MAC 5000 device (Marquette Electronics, Inc., Milwaukee, Wisconsin). The QT interval measurements were made using ECG Interval Editor analysis software version 005D.04 (General Electric HC, Menomonee Falls, Wisconsin) which has demonstrated good correlation with other automated programs and manual measurements [22].

In the ARIC study, participants were asked not to smoke or ingest caffeine for at least 1 hour prior to the electrocardiogram. After resting for 5–10 minutes while the electrodes were being placed, a standard supine 12-lead electrocardiogram and a 2-minute paper recording of a three-lead (leads V_1_, II, and V_5_) rhythm strip were made. The ECGs were digitally recorded, and identical methods (MAC personal computer, Marquette Electronics, Milwaukee, Wisconsin) were used in all clinical centers. A similar protocol was used at the baseline visit of CHS. MAC PC-DT ECG acquisition units (Marquette Electronics, Inc., Milwaukee, WI) were used to record a 10-second 12-lead simultaneous ECG at a sample rate of 250 per second per lead. The QT interval from the digital 12-lead ECG was determined using the Novacode ECG measurement and classification program [23].

### SNP Selection and Genotyping

SNPs were selected to tag the linkage disequilibrium (LD) block containing rs10494366 and rs4657139 (the most significant SNPs from fine mapping in our previous studies in whites [9]) in the thirty trio samples of U.S. residents with northern and western European ancestry (CEU population) used in the HapMap Project [24], [25]. Eight were selected using the computer program Tagger with criteria of r^2^>0.65 and minor allele frequency (MAF) >0.05 in CEU [26].

Genotyping in the DHS was performed using ABI TaqMan assays (Applied Biosystems) according to standard protocols. Genotyping in ARIC and CHS was performed using TaqMan assays (Applied Biosystems) in conjunction with the BioTrove OpenArray SNP genotyping platform, which incorporates high-density, through-hole, nanotiter plates which have 3,072 holes penetrating the slide, each of which is suitable for a TaqMan assay (www.biotrove.com). Accuracy of BioTrove genotyping was determined by comparison to concordance calls generated for 58 samples genotyped multiple times (range 2–19 times, median 6, resulting in ∼350 comparison per SNP): rs16847548 = 99.7%; rs16856785 = 99.4%.

### Statistical Analysis

All analyses were stratified by self-reported ethnicity. Deviations from Hardy-Weinberg proportions were assessed using the chi-squared goodness of fit test within each ethnicity group. For the DHS, all QT interval results were generated using the residuals from race-specific linear regressions adjusted for age, sex, and heart rate (RR interval). A generalized linear model was then used to assess the association between SNPs and residual QT intervals assuming an additive genetic model. In the ARIC/CHS cohort, analyses were performed using linear regression adjusted for age, sex, heart rate (RR interval), and study. Parallel analyses were also performed using Bazett's heart rate-corrected QT duration (QTc) [27], and similar results and inferences were obtained (data not shown). Forward step-wise regression analysis was performed by first incorporating the SNP with most significant association from single-SNP analyses, and sequentially adding SNPs in order of strength of association, and only retaining a SNP in the model if P-values were <0.05. To test for interaction between SNPs and sex, we used linear regression with terms in the model for the SNP (under an additive model), sex, and SNP × sex interaction term, with the significance reported for the interaction term. All statistical analyses were performed in R version 2.6.2.
